# Crystal Structure of the Receptor-Binding Domain of Botulinum Neurotoxin Type HA, Also Known as Type FA or H

**DOI:** 10.3390/toxins9030093

**Published:** 2017-03-08

**Authors:** Guorui Yao, Kwok-ho Lam, Kay Perry, Jasmin Weisemann, Andreas Rummel, Rongsheng Jin

**Affiliations:** 1 Department of Physiology & Biophysics, University of California, Irvine, CA 92697, USA; g.yao@uci.edu (G.Y.); kwokhl@uci.edu (K.-h.L.); 2 NE-CAT and Department of Chemistry and Chemical Biology, Cornell University, Building 436E, Argonne National Laboratory, 9700 S. Cass Avenue, Argonne, IL 60439, USA; kperry@anl.gov; 3 Institut für Toxikologie, Medizinische Hochschule Hannover, Carl-Neuberg-Str. 1, 30625 Hannover, Germany; weisemann.jasmin@mh-hannover.de (J.W.); rummel.andreas@mh-hannover.de (A.R.)

**Keywords:** botulinum neurotoxin (BoNT), BoNT/HA, BoNT/H, BoNT/FA, receptor-binding domain, host receptor, neutralizing antibody

## Abstract

Botulinum neurotoxins (BoNTs), which have been exploited as cosmetics and muscle-disorder treatment medicines for decades, are well known for their extreme neurotoxicity to humans. They pose a potential bioterrorism threat because they cause botulism, a flaccid muscular paralysis-associated disease that requires immediate antitoxin treatment and intensive care over a long period of time. In addition to the existing seven established BoNT serotypes (BoNT/A–G), a new mosaic toxin type termed BoNT/HA (aka type FA or H) was reported recently. Sequence analyses indicate that the receptor-binding domain (H_C_) of BoNT/HA is ~84% identical to that of BoNT/A1. However, BoNT/HA responds differently to some potent BoNT/A-neutralizing antibodies (e.g., CR2) that target the H_C_. Therefore, it raises a serious concern as to whether BoNT/HA poses a new threat to our biosecurity. In this study, we report the first high-resolution crystal structure of BoNT/HA-H_C_ at 1.8 Å. Sequence and structure analyses reveal that BoNT/HA and BoNT/A1 are different regarding their binding to cell-surface receptors including both polysialoganglioside (PSG) and synaptic vesicle glycoprotein 2 (SV2). Furthermore, the new structure also provides explanations for the ~540-fold decreased affinity of antibody CR2 towards BoNT/HA compared to BoNT/A1. Taken together, these new findings advance our understanding of the structure and function of this newly identified toxin at the molecular level, and pave the way for the future development of more effective countermeasures.

## 1. Introduction

Botulism is a rare but life-threatening disease caused by botulinum neurotoxins (BoNTs), one of the most poisonous natural substances known. BoNTs are categorized as a Tier 1 select agent by the Centers for Disease Control and Prevention (CDC) and could be potentially misused for bioterrorism warfare [1,2]. Paradoxically, some BoNTs have been successfully used as prescription medicines to treat muscle disorders or as injectable facial rejuvenation agents. Naturally occurring botulism forms are mostly food-borne botulism and infant botulism [3,4], in which the toxins are absorbed in the intestine and colon, respectively, into the general circulation. BoNTs specifically target neuromuscular junctions (NMJ), where the toxins are internalized into neuronal cells, cleave the soluble *N*-ethylmaleimide sensitive factor attachment protein receptors (SNAREs) complex, inhibit the release of neurotransmitter acetylcholine, and eventually paralyze the affected muscles [5,6].

BoNTs are large proteins (~150 kDa), which are produced in bacteria in the form of progenitor toxin complexes (PTCs, 300–760 kDa) that are composed of BoNT and several non-toxic neurotoxin-associated proteins (NAPs) [7,8,9,10,11,12]. Structurally, BoNT consists of an N-terminal light chain (LC, ~50 kDa) that is a metalloprotease and a C-terminal heavy chain (HC, ~100 kDa). The latter could be further divided into two domains: the N-terminal portion (~50 kDa) is the translocation domain (H_N_) required for the LC to be released into the cytosol; the C-terminal part (H_C_) is the receptor-binding domain responsible for the highly specific binding and endocytosis of BoNT into motoneurons. Among the seven established serotypes of BoNT (BoNT/A-G) [13], more than 40 BoNT subtypes have been identified [14,15] (e.g., BoNT/A1–A8). In addition, two hybrid/mosaic types have been identified: BoNT/CD and BoNT/DC. BoNT/DC comprises a LC and a H_N_ domain highly homologous to BoNT/D and a H_C_ similar to BoNT/C, whereas BoNT/CD closely resembles BoNT/C in the LC and H_N_ domains but shares a high sequence similarity with BoNT/D in the H_C_ domain [14,16,17].

A new BoNT type, originally termed BoNT/H, was reported in 2014 [18,19]. This toxin was produced in the bivalent *Clostridium botulinum* strain IBCA10-7060, which also expresses BoNT/B2 [18,19,20]. This new toxin was later successfully separated from BoNT/B2 in native host strain by inactivating the *bont*/*B2* gene [21]. This toxin was originally categorized as a new serotype because the strain supernatant failed to be neutralized by several existing antitoxins including a US Army-supplied equine heptavalent F(ab’)_2_ botulinum antitoxin A–G at a testing ratio as high as 595:1 (antitoxin:toxin) [18]. Subsequent studies showed that a licensed, commercially available antitoxin, BAT (Botulism Antitoxin Heptavalent (A, B, C, D, E, F, G)—Equine), was able to neutralize this newly identified toxin [20]. Furthermore, several polyclonal antibodies raised against BoNT/A1 were found to neutralize this toxin, but at a lower potency compared to BoNT/A1 [21]. A new potent monoclonal antibody directed against this new toxin was reported in 2016 [22].

Amino acid (AA) sequence alignments based on genome sequences of strain IBCA10-7060 [19,23] showed that the H_C_ of this toxin is most similar to that of BoNT/A1 (~84% identity), while its LC and H_N_ share ~81% and ~64% identity to that of BoNT/F5, respectively [20]. An alternative nomenclature of this toxin as BoNT/FA was then proposed [23]. Subsequently, it was confirmed that this new toxin cleaves VAMP-2 (also called synaptobrevin 2) between L54 and E55 [21,24], which is identical to the behavior of BoNT/F5, but different from all other BoNT/F subtypes that cut VAMP-2 between Q58 and K59 [25]. Interestingly, only one of the six anti-BoNT/F antibodies tested in a recent study showed binding to the new toxin, albeit weak (K_D_ ~75 nM), suggesting that it is immunologically different from BoNT/F [22]. In contrast, monoclonal antibodies RAZ1 and CR2 (both target the H_C_ of BoNT/A) precipitated the new toxin from the culture supernatant [24] and neutralized the new toxin in a mouse bioassay [22]. Based on these data, we suggested to name this new toxin as BoNT/HA [26].

Currently no consensus about the nomenclature of this new toxin has been reached [15]. The scientific debates on this topic are largely due to the lack of understanding of its structure and function at the molecular level. Most of the current studies on BoNT/HA have been based on genomic and amino acid sequence analysis. This approach has been proven erroneous in the case of a mosaic BoNT/DC, whose H_C_ is very similar to BoNT/C based on sequence (~64% identity). However, the crystal structure of BoNT/DC clearly shows that its H_C_ is more similar to BoNT/B (~22% identity) than BoNT/C, which has been confirmed by functional studies [17,27]. Therefore, a crystal structure of BoNT/HA is essential to compare it with the other known BoNTs at the molecular level.

In this paper, we focused our study on the H_C_ that is a proven target for anti-BoNT antibody and vaccine development. In our previous study, we showed that the H_C_ of BoNT/HA (H_C_HA) binds weakly to the protein moiety of its cell-surface receptor SV2C when compared to H_C_A1 in spite of high sequence similarity [26]. Furthermore, a highly potent anti-BoNT/A antibody CR2, which is currently in clinical trials, displayed a ~540-fold decreased affinity on BoNT/HA according to a recent neutralization study [22]. These findings thus raised questions on how BoNT/HA behaves differently than BoNT/A1 on neuronal receptor binding and responds differently to the known antitoxins. To gain a better understanding of the structure and function of BoNT/HA, we determined the crystal structure of H_C_HA at a high resolution.

## 2. Results and Discussion

### 2.1. Biochemical Characterization of H_C_HA

Sequence alignment showed that H_C_HA shares ~84% AA sequence identity to H_C_A1. While the H_CN_ part is more variable between the two (~75%), the H_CC_ portion is highly conserved (~93%). The H_CC_ of BoNT/A bears important binding sites for two cell-surface receptors: polysialogangliosides (PSGs) and synaptic vesicle glycoprotein 2 (SV2, including three isoforms SV2A, SV2B, and SV2C), which are required for the extreme neurotoxicity of the toxin according to the dual-receptor binding model [28]. Given the high sequence similarity, it is not surprising that BoNT/HA also uses SV2 as its protein receptor [26]. We successfully expressed and purified H_C_HA (residues E860-L1288) and H_C_A1 (residues N872-L1296) using *Escherichia coli*. Interestingly, we noticed that H_C_HA has a significantly lower yield of expression than H_C_A1 and also has a very low solubility, ~1 mg/mL in a buffer that contains 150 mM NaCl at pH 7.5. In contrast, H_C_A1 could be concentrated to at least 15 mg/mL under the same condition. After large-scale crystallization screens, H_C_HA was successfully crystallized at 1 mg/mL, an unusually low protein concentration for protein crystallization in general.

Via sequence alignment we noticed that an arginine of H_C_A1 (R1156) was substituted by a methionine in H_C_HA (M1148). The solubility of H_C_HA was significantly increased when we introduced a single-point mutation (M1148R) to H_C_HA, which could be concentrated to over 8 mg/mL in the same buffer mentioned above. On the other hand, a reversed mutation on H_C_A1 (R1156M) severely decreased its solubility and also significantly hampered the expression yield of H_C_A1 in *E. coli*. In spite of low solubility in solution, H_C_HA is correctly folded as the protein-melting assay showed that the thermo-stability of H_C_HA is slightly better than H_C_A1 (59.9 °C for H_C_A1 and 62.7 °C for H_C_HA) [26].

### 2.2. The Crystal Structure of H_C_HA

**Figure 1:**
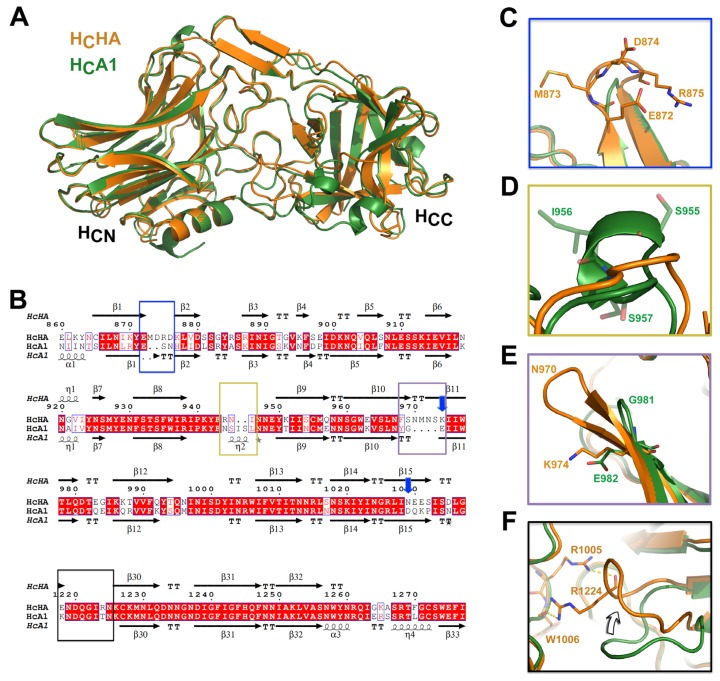
Sequence alignment and structural comparison between H_C_HA and H_C_A1. (**A**) Superimposition of the structures of H_C_HA and H_C_A1. (**B**) Amino acid (AA) sequence alignment of H_C_HA and H_C_A1. Secondary structures of H_C_HA and H_C_A1 are placed on the top and the bottom, respectively. Blue arrows indicate the known pH-sensing residues on BoNT/A1 [11]. The four boxes highlight major AA sequence variations between H_C_HA and H_C_A1 that lead to structural changes. Close-up views of the structures in the corresponding areas are shown in (**C**) the blue box, (**D**) the yellow box, (**E**) the purple box, and (**F**) the black box. The AA sequence of H_C_A1 and H_C_HA are taken from GenBank: AAQ06331.1 (H_C_A1) and KGO15617.1 (H_C_HA). Sequence alignments were performed using Cluster Omega 1.2.2 and displayed with ESPript 3.0 [30,31].

We then determined the crystal structure of H_C_HA at 1.8 Å resolution (Table 1). The overall structure of H_C_HA and H_C_A1 are very similar with a root-mean-square (RMS) deviation of 0.526 Å over 341 Cα atoms (Pymol, www.pymol.org). Close inspection of the structures of H_C_HA and H_C_A1 (PDB 5JLV) revealed several unique structural features of H_C_HA [26]. First, the short α-helix (residues N872-T876) at the very N-terminus of H_C_A1 is unstructured in H_C_HA (Figure 1A,B). Second, the loop (residues M873-K877) that links the first and the second β-strand in H_C_HA is longer than that in H_C_A1 (residues S885-H887) because H_C_HA has two extra residues in this region (Figure 1B, blue box; Figure 1C). Interestingly, this loop is not conserved among BoNT/A subtypes. In BoNT/A8, this loop is longer than that in other subtypes because of a unique arginine insertion that makes BoNT/A8 the longest BoNT/A subtype [29]. The third notable difference is that a 3/10-helix (residues S955-S957) linking β8 and β9 in H_C_A1 is unstructured in H_C_HA due to the deletion of two residues in this region (Figure 1B, yellow box; Figure 1D).

We noticed that the β10 and β11 of H_C_HA are longer than those of H_C_A1 due to the insertion of four more residues (residues N970-S973) (Figure 1B, purple box; Figure 1E). Interestingly, this region of BoNT/A1 is involved in the release of BoNT/A1 from the PTC during oral intoxication. Earlier studies suggest that an auxiliary bacterial protein, non-toxic non-hemagglutinin (NTNHA), binds and protects BoNT/A1 in the gastrointestinal tract, and that BoNT/A1 is released from NTNHA upon absorption due to environmental pH change [7,11]. Notably, residue E982 located on β11 of H_C_A1 was reported as one of the pH sensing residues, which are deprotonated upon absorption and subsequently trigger the dissociation of BoNT/A1 from its NTNHA [11]. This residue is highly conserved in all BoNT/A subtypes. However, H_C_A1-E982 is replaced by a lysine in H_C_HA (K974). Interestingly, another pH sensing residue on H_C_A1, D1037, is also conserved in all BoNT/A subtypes, but differs from that of BoNT/HA (N1029) (Figure 1B, blue arrow) [11]. Nevertheless, BoNT/HA could be released from the M-PTC at neutral pH, as evidenced by the separation of BoNT/HA from NTNHA that is encoded in its operon at pH 7.6 [21]. Thus, BoNT/HA may employ a different set of pH sensing residues than BoNT/A to release it from the M-PTCs.

Another major structural difference between H_C_HA and H_C_A1 is found in the loop that links β29 and β30 (Figure 1B, black box; Figure 1F). In H_C_HA this loop (residues E1218-K1226) swings ~45 degrees towards the center of the H_CC_ subdomain compared to that of H_C_A1. This is likely caused by residue H_C_HA-R1224 (equivalent to H_C_A1-T1232), which forms multiple hydrogen bonds with residues R1005 and W1006. The physiological relevance of this conformational difference on H_C_HA is currently unknown.

### 2.3. H_C_HA and H_C_A1 Bind Differently to Cell-Surface Receptors

**Figure 2:**
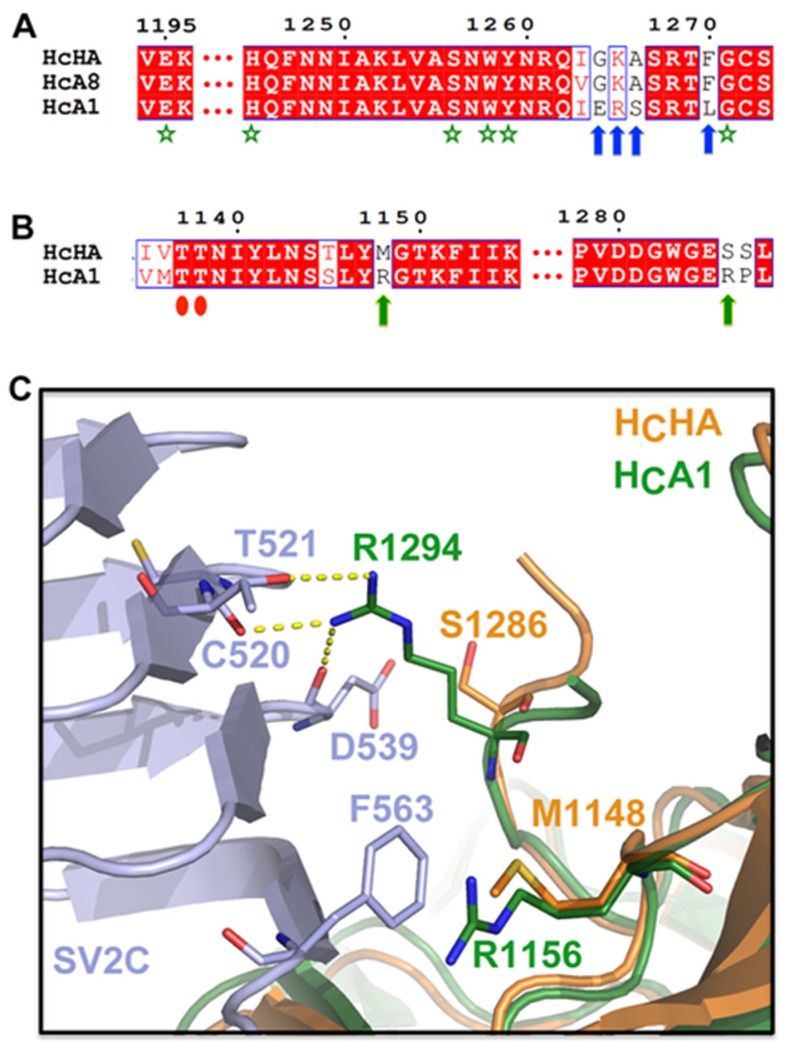
Genetic changes between BoNT/HA and BoNT/A1 lead to different receptor-binding modes. (**A**) A sequence alignment suggests that the ganglioside-binding mode of BoNT/HA closely resembles that of BoNT/A8 and slightly differs from BoNT/A1. The conserved ganglioside-binding site (GBS) motif is indicated by green stars. Blue arrows highlight residues of H_C_HA that are identical to H_C_A8 but different from H_C_A1. The AA sequence of H_C_A1, H_C_A8, and H_C_HA are taken from GenBank: AAQ06331.1, AJA05787.1, and KGO15617.1. (**B**) A sequence alignment shows that two H_C_A1 residues, R1156 and R1294 (green arrows), whose side chains are engaged in SV2C binding, are not conserved in H_C_HA. Red ovals indicate two conserved residues that mediate backbone-backbone interactions between H_C_HA/H_C_A1 and SV2C. (**C**) Superimposition of the structures of H_C_HA (PDB 5V38) and the SV2C-H_C_A1 complex (PDB 5JLV) reveals the missing cation-π interaction and hydrogen bondings when H_C_HA binds to SV2C.

According to a well-accepted dual-receptor binding model [28,32,33], BoNTs bind to neuronal cell surface through subsequent associations with two receptors: polysialogangliosides and a protein receptor. Sequence analysis and structural alignment show that the ganglioside-binding site (GBS) motif ‘E…H…SXWY…G’, which is highly conserved in BoNT/A, B, E (residue H is replaced by a K), F, and G (residue E is replaced by a Q) [34], is strictly conserved in H_C_HA (E1195, H1245, S1256, W1258, Y1259, and G1271) (Figure 2A, green stars). Interestingly, our sequence alignment shows that the H_CC_ of BoNT/HA is most similar to BoNT/A8 (~95%). The alignment also suggests that a surface-exposed loop in the GBS neighborhood on BoNT/HA (residues I1263-A1266) is highly similar to BoNT/A8 (residues V1272-A1275), but differs from BoNT/A1 (residues I1271-S1274) (Figure 2A, blue arrows). This region of H_C_HA is likely flexible because the electron densities from residues R1261-R1268 were missing in our structure. Nevertheless, it is reasonable to speculate that the ganglioside-binding profile of BoNT/HA is more similar to BoNT/A8 than BoNT/A1. It is noteworthy that H_C_A8 has a weaker binding affinity to gangliosides on neuronal membranes compared to H_C_A1, which is possibly due to the negative influence of this altered loop [29]. Thus, we suspect that the reduced ganglioside-binding ability of BoNT/HA could partly contribute to its ~5-fold lower toxicity compared to BoNT/A1, as revealed by a mouse bioassay [21].

We have shown previously that BoNT/HA also uses SV2 as its cell-surface protein receptor [26]. The new structure of H_C_HA revealed that the subtle amino acid differences between BoNT/HA and BoNT/A actually lead to their substantially different protein-protein interactions with SV2. For example, an arginine on H_C_A1 (R1156) that contributes a crucial cation-π interaction with SV2C-F563 is replaced by a methionine (M1148) on H_C_HA (Figure 2B,C). Interestingly, this residue is not conserved amongst BoNT/A subtypes [26]. Apparently, the neighboring H_C_HA-R1134 cannot take over the cation-π interaction with SV2C-F563. Furthermore, while H_C_A1-R1294 forms three hydrogen bonds with C520, T521, and D539 of SV2C through its long, well-extended side-chain (Figure 2B,C), these hydrogen bondings are likely missing on H_C_HA because H_C_A1-R1294 is replaced by a serine (H_C_HA-S1286). These findings thus explain the observation that H_C_A1 was able to bind the non-glycosylated human SV2C-L4 (luminal domain 4), while H_C_HA showed a clearly decreased binding.

**Figure 3:**
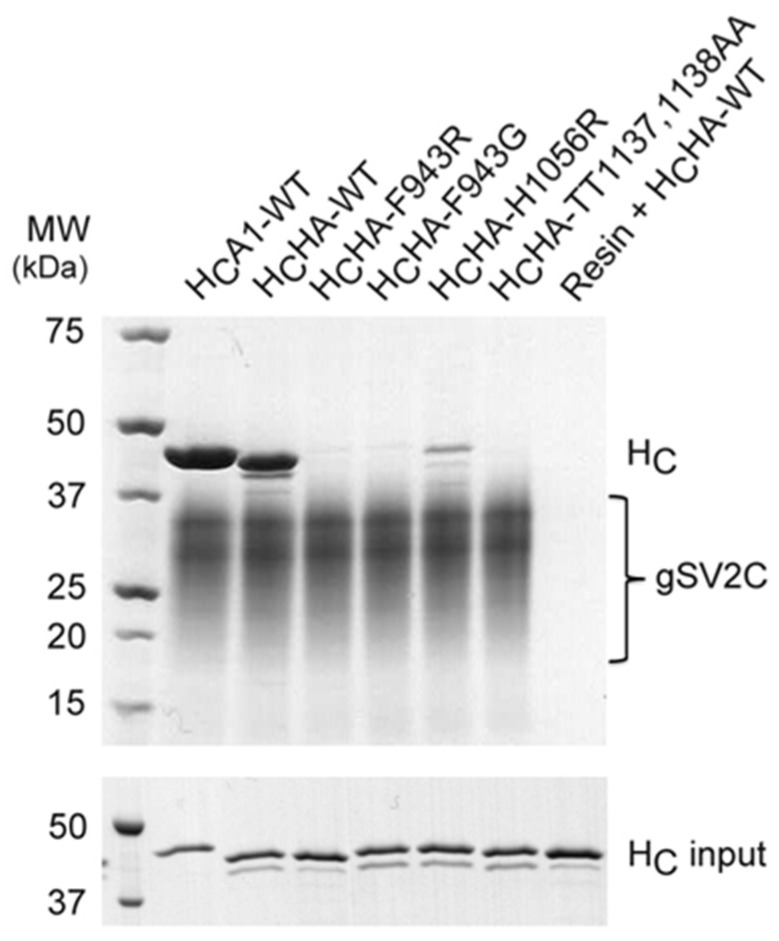
Protein-glycan interactions play an important role in BoNT/HA-SV2C recognition. The pulldown assay was performed to examine interactions between the glycosylated SV2C (gSV2C, bait) and H_C_A1 or H_C_HA variants (preys).

In spite of the weak protein-based interactions, H_C_HA binds strongly to the glycosylated SV2C (gSV2C), which clearly emphasizes the important role of SV2 glycan for BoNT/HA binding (Figure 3). Residues F953 and H1064 of H_C_A1 that form strong π-stacking interactions with the N559 glycan of gSV2C are conserved on H_C_HA (F943 and H1056). Disrupting the π-stacking interaction by introducing single point mutations on H_C_HA (e.g., F943R, F943G, and H1056R) showed strong reduction of binding to gSV2C based on a pull-down assay (Figure 3). Nevertheless, protein-protein interactions between H_C_HA and gSV2 are indispensable [26,35]. Disrupting two crucial backbone-backbone hydrogen bonds between H_C_HA and gSV2 (H_C_HA-T1137A/T1138A) led to dramatically decreased binding of H_C_HA to gSV2C (Figure 3).

Interestingly, a recent study showed that BoNT/HA was ~4.3- and ~15-fold more active than BoNT/A1 when cleavage of VAMP-2 was examined using cultured primary rat spinal cord cells and human induced pluripotent stem cells (hiPSC)-derived neurons, respectively [21]. We suspect that the different potency of BoNT/HA revealed by cultured neuron-based assay and mouse bioassay could be partly caused by different tissue distribution of the three SV2 isoforms and potentially different N-linked glycosylation at N559. For example, SV2C is hardly present in cortical neurons [36,37,38], but dominant in motoneurons and spinal cord neurons, whereas SV2A is the dominant isoform in cortical neurons and the hiPSC-derived neurons [39]. Further research is needed to address these differences.

### 2.4. BoNT/HA and BoNT/A1 Respond Differently to Existing Antibodies

**Figure 4:**
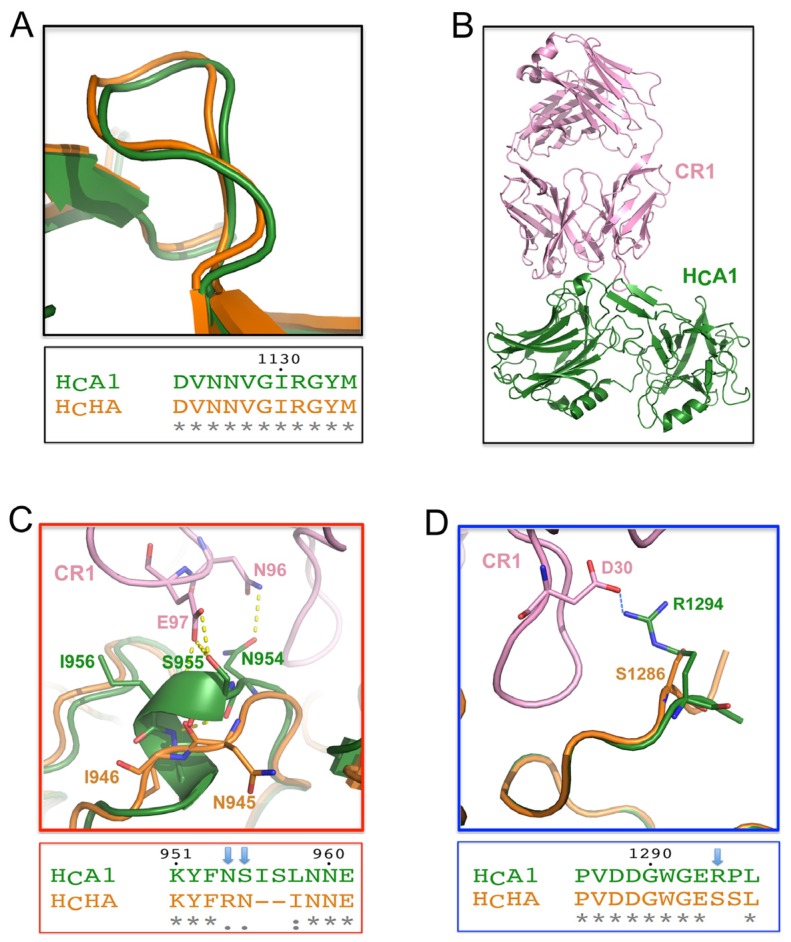
Binding of BoNT/HA to two potent BoNT/A1-neutralizing antibodies. (**A**) The structure and sequence at the RAZ1 binding site are identical between H_C_HA and H_C_A1. (**B**) Overall structure of CR1 in complex with H_C_A1 (PDB 2NYY). Superimposition of the structures of H_C_HA and the CR1-BoNT/A1 complex shows that: (**C**) some hydrogen bonds at a 3/10 helix of H_C_A1 are missing on H_C_HA, and (**D**) a crucial salt bridge at the C-terminus of H_C_A1 is missing on H_C_HA.

Antibody neutralization is currently the most effective way to counteract BoNTs. Due to the high degree of AA sequence identity between H_C_HA and H_C_A, it was expected that antibodies that target H_C_A are likely able to neutralize BoNT/HA and thus reduce this new threat to society. A recent study found that several antibodies could work in this regard, including RAZ1 and CR2, which are two of the three antibodies in the clinical-trial drug XOMA 3AB [40]. It was reported that RAZ1 (derived from 3D12) bound BoNT/HA tightly with a dissociation constant (K_D_) of ~4.96 pM, almost as good as BoNT/A1 (~2 pM) [22]. The binding epitope of 3D12 on BoNT/A1 has been mapped to residues N1127-R1131 [41,42]. This region is identical between BoNT/A1 and BoNT/HA (Figure 4A), thus explaining the high potency of RAZ1 on BoNT/HA.

Even though the overall structures of H_C_HA and H_C_A are highly similar, CR2 showed a surprisingly ~540-fold lower binding affinity to BoNT/HA (K_D_ ~5.37 nM) than BoNT/A1 (K_D_ ~10 pM) [22]. Since the structure of CR2 is not available, we used the crystal structure of a closely related antibody CR1 for analyses (Figure 4B), which differs from CR2 at only two residues (E6Q and V37I) that are far away from the BoNT/A1-binding interface [43]. CR1/CR2 efficiently neutralize BoNTA1 by occupying the SV2-binding site on the toxin [26]. We found that some crucial interactions between H_C_A1 and CR1/CR2 are missing on H_C_HA due to genetic changes. For example, while a 3/10-helix of H_C_A1 (residues S955-S957) and residue N954 form multiple hydrogen bonds with CR1/CR2 (through residues N96 and E97 of CR1), these polar contacts are missing on H_C_HA, which is very different from H_C_A in this region (Figure 4C). Furthermore, an important salt bridge between H_C_A1-R1294 and CR1-D30 is missing on H_C_HA due to the replacement of arginine by a serine on H_C_HA (H_C_HA-S1286) (Figure 4D). Taken together, these data suggest that BoNT/HA is able to escape from highly potent BoNT/A-neutralizing CR2 due to subtle genetic changes.

## 3. Conclusions

In summary, we have successfully expressed and purified the recombinant receptor-binding domain of the newly discovered botulinum neurotoxin type HA (H_C_HA). Dramatically different solubility in solution was observed between H_C_HA and H_C_A1, which was largely caused by variation of a single residue (H_C_A1-R1156 and H_C_HA-M1148). We then crystallized H_C_HA and determined its 3-dimensional structure at 1.8 Å resolution. Systematic sequence alignment and structural comparison between H_C_HA and H_C_A1 revealed many unique features of BoNT/HA. In brief, we found that BoNT/HA may use a different set of residues than BoNT/A to sense the environmental pH change in order to be released from the M-PTC during absorption in the gastrointestinal tract. Furthermore, BoNT/HA presumably displays different interactions with neuron-surface gangliosides. While the peptidic interactions of the protein receptor SV2C are weaker with BoNT/HA than BoNT/A1, the binding of BoNT/HA to the N559-glycan of SV2C compensates for this deficit. Importantly, the novel structure of H_C_HA also provides clear explanations to the high potency of RAZ1 and ~540-fold decreased potency of CR2 against BoNT/HA versus BoNT/A1. Taken together, the new structural insights into the neuron-binding mode of BoNT/HA and how it dampens binding of some existing BoNT/A neutralizing antibodies will facilitate further research exploring the function of BoNT/HA, and also help the development of more specific and potent antibodies against BoNT/HA as new research tools and potential therapeutic agents.

## 4. Materials and Methods

### 4.1. Plasmid Construction

H_C_HA (residues E860-L1288 of BoNT/HA) was cloned into pGEX-4T-2 vector with an N-terminal glutathione S-transferase (GST) and a thrombin cleavage site. H_C_A1 (residues N872-L1296 of BoNT/A1) following a PreScission protease cleavage site was cloned into pQE30 vector with an N-terminal His_6_-tag. H_C_HA point mutations were generated with QuikChange site-directed mutagenesis (Agilent, Santa Clara, CA, USA). The sequence corresponding to the core region of human SV2C-L4 (residues V473-T567) was cloned into pcDNA vector for mammalian expression. A human IL2 signal sequence (MYRMQLLSCIALSLALVTNS) and a His_9_-tag followed by a Factor Xa cleavage site were added to the N-terminus of SV2C.

### 4.2. Protein Expression and Purification

H_C_HA (wild-type and mutants) and H_C_A1 were expressed in *E. coli* strain BL21-Star (DE3) (Invitrogen, Carlsbad, CA, USA). Bacteria were cultured at 37 °C in LB (Luria-Bertani) medium containing appropriate selecting antibiotics. The temperature was set to 18 °C when OD_600_ reached 0.4. For induction, IPTG (isopropyl-b-d-thiogalactopyranoside) with a final concentration of 0.2 mM was added to the culture when OD_600_ reached 0.7. The expression was continued at 18 °C for 16 h after induction. The cells were harvested by centrifugation.

H_C_HA was purified using Glutathione Sepharose 4B affinity beads (GE Healthcare Life Sciences, Pittsburgh, PA, USA) in a buffer containing 50 mM Tris, pH 8.0; 400 mM NaCl. H_C_HA was released from the beads by on-column cleavage at 4 °C using thrombin. H_C_A1 was purified using Ni-NTA (Qiagen, Germantown, MD, USA) affinity resins in the same buffer supplemented with 40 mM imidazole and subsequently eluted with a high-imidazole buffer (50 mM Tris, pH 8.0; 400 mM NaCl; 300 mM imidazole). The proteins were then dialyzed at 4 °C against a buffer containing 20 mM HEPES, pH 7.5; 150 mM NaCl. The His_6_-tag of H_C_A1 was then excised by human rhinovirus 3C protease.

Tag-cleaved H_C_HA and H_C_A1 were further purified by MonoS ion-exchange chromatography (GE Healthcare Life Sciences, Pittsburgh, PA, USA) in a buffer containing 50 mM MES, pH 6.0 and eluted with a NaCl gradient. The peak fractions were then subjected to Superdex 200 size-exclusion chromatography (SEC, GE Healthcare Life Sciences, Pittsburgh, PA, USA) in a buffer containing 20 mM HEPES, pH 7.5, and 150 mM NaCl. Peak fractions were collected and concentrated for the downstream experiments.

Human SV2C-L4-core (gSV2C) was expressed and secreted from HEK 293 cells and purified directly from medium using Ni-NTA resins. The protein was eluted from the resins using high concentration of imidazole and dialyzed against a buffer containing 50 mM Tris, pH 8.0; 400 mM NaCl. gSV2C was then subjected to Superdex 200 SEC in a buffer containing 20 mM HEPES, pH 7.5; 150 mM NaCl.

### 4.3. Pull-Down Assay

The pull-down assay was performed using Ni-NTA resins in 1 mL buffer containing 50 mM Tris, pH 8.0; 400 mM NaCl; 10 mM imidazole and 0.1% Tween-20. gSV2C was used as the bait while H_C_A1-wildtype, H_C_HA-wildtype and mutants were preys. gSV2C was pre-incubated with Ni-NTA resins at 4 °C for 1 h. After the unbound protein was washed away, the resins were divided into small aliquots. Roughly, ~5 μg of gSV2C (~3 μM) was bound on the resins in each tube, and 30 μg of H_C_ (~6 μM) was then added. The resins were washed twice after ~1.5 h incubation at 4 °C. The bound proteins and H_C_ inputs were released from the resins using a SDS sample buffer containing 300 mM imidazole, and visualized by SDS-PAGE. All experiments were carried out in parallel for direct comparison.

### 4.4. Crystallization

Initial crystallization screens of H_C_HA were carried out using a Gryphon crystallization robot (Art Robbins Instrument, Sunnyvale, CA, USA) with high-throughput crystallization screening kits (Hampton Research, Aliso Viejo, CA, USA and Qiagen, Germantown, MD, USA). After extensive manual optimizations, single crystals were grown at 18 °C by the hanging-drop vapor-diffusion method using a 1:1 (*v*/*v*) ratio of protein and the reservoir (100 mM sodium acetate, pH 4.2, and 20% Polyethylene glycol (PEG) 3350). The crystals were cryoprotected in the original mother liquor supplemented with 20% (*v*/*v*) glycerol and flash-frozen in liquid nitrogen.

### 4.5. Data Collection and Structure Determination

Crystals were screened at SSRL and NE-CAT. The best diffraction data were collected at the NE-CAT beamline 24-ID, Advanced Photon Source (APS), Argonne, IL, USA. The data were processed with iMosflm [44]. The H_C_HA structure was determined with Phaser molecular replacement [45] using the structure of H_C_A1 (PDB code 5JLV) as the search model. The structural modeling and refinement were carried out iteratively using COOT [46] and Refmac from the CCP4 suite [47]. The refinement progress was monitored with the R_free_value with a 5% randomly selected test set [48]. The structure was validated by the MolProbity web server [49], and the refinement statistics are listed in Table 1. All structure figures were prepared with PyMOL (http://www.pymol.org/).

### 4.6. Accession Code

Coordinates and structure factors for H_C_HA have been deposited in the Protein Data Bank under accession code 5V38.

## Figures and Tables

**Table 1:** Data collection and refinement statistics.

	H_C_HA (PDB ID 5V38)
**Data collection**	
Space group	P 1 21 1
Cell dimensions	
*a*, *b*, *c* (Å)	77.94; 80.12; 78.38
α, β, γ (°)	90.00°; 105.94°; 90.00°
Resolution (Å)	49.23–1.80 (1.83–1.80) ^a^
*R*_meas_	0.123 (0.723)
*I/*σ(*I*)	8.1 (2.2)
*CC*_1/2_	0.995 (0.646)
Completeness (%)	97.5 (97.0)
Redundancy	3.3 (3.3)
**Refinement**	
Resolution (Å)	49.23–1.80 (1.83–1.80)
No. reflections	83495
*R*_work_/*R*_free_	0.179/0.217
No. atoms	
Protein	834
Ligand	7
Water	926
*B* factors	
Protein	21.00
Ligand	35.00
Water	33.80
RMS deviations	
Bond lengths (Å)	0.007
Bond angles (°)	1.00

^a^ Values in parentheses are for the highest-resolution shell. RMS, root-mean-square.
